# Prognosis and Immunotherapy Significances of a Cancer-Associated Fibroblasts-Related Gene Signature in Gliomas

**DOI:** 10.3389/fcell.2021.721897

**Published:** 2021-10-29

**Authors:** Zhimin Chen, Shenghua Zhuo, Guiying He, Jingzhi Tang, Weijie Hao, Wei-Qiang Gao, Kun Yang, Huiming Xu

**Affiliations:** ^1^State Key Laboratory of Oncogenes and Related Genes, Renji-MedX Clinical Stem Cell Research Center, Ren Ji Hospital, School of Medicine, Shanghai Jiao Tong University, Shanghai, China; ^2^Department of Neurosurgery, The First Affiliated Hospital of Hainan Medical University, Haikou, China; ^3^Department of Neurology, Shenzhen Nanshan People’s Hospital, The 6th Affiliated Hospital of Shenzhen University Health Science Center, Shenzhen, China

**Keywords:** glioma, cancer-associated fibroblasts, tumor microenvironment, immunotherapy, prognosis, risk score

## Abstract

As a cold tumor, malignant glioma has strong immunosuppression and immune escape characteristics. The tumor microenvironment (TME) provides the “soil” for the survival of malignant tumors, and cancer-associated fibroblasts (CAFs) are the architects of matrix remodeling in TME. Therefore, CAFs have potent regulatory effects on the recruitment and functional differentiation of immune cells, whereby they synthesize and secrete numerous collagens, cytokines, chemokines, and other soluble factors whose interaction with tumor cells creates an immunosuppressive TME. This consequently facilitates the immune escape of tumor cells. Targeting CAFs would improve the TME and enhance the efficacy of immunotherapy. Thus, regulation of CAFs and CAFs-related genes holds promise as effective immunotherapies for gliomas. Here, by analyzing the Chinese Glioma Genome Atlas and the Cancer Genome Atlas database, the proportion of CAFs in the tumor was revealed to be associated with clinical and immune characteristics of gliomas. Moreover, a risk model based on the expression of CAFs-related six-gene for the assessment of glioma patients was constructed using the least absolute shrinkage and selection operator and the results showed that a high-risk group had a higher expression of the CAFs-related six-genes and lower overall survival rates compared with those in the low-risk group. Additionally, patients in the high-risk group exhibited older age, high tumor grade, isocitrate dehydrogenase wildtype, 1p/19q non-codeletion, O-6-methylguanine-DNA methyltransferase promoter unmethylation and poor prognosis. The high-risk subtype had a high proportion CAFs in the TME of glioma, and a high expression of immune checkpoint genes. Analysis of the Submap algorithm indicated that the high-risk patients could show potent response to anti-PD-1 therapy. The established risk prediction model based on the expression of six CAFs-related genes has application prospects as an independent prognostic indicator and a predictor of the response of patients to immunotherapy.

## Introduction

Glioma is the most common form of a primary malignant brain tumor in adults. In particular, glioblastoma (GBM) is one of the most lethal and highly aggressive cancer, associated with low survival, less than 1 year (Lapointe et al., 2018). Although current evidence indicates surgical resection as the main treatment approach for gliomas, postoperative radiotherapy, and chemotherapy can also be administered according to the specific condition of patients. However, most gliomas are difficult to completely be removed by surgery without affecting normal brain functions due to the growth properties and its special local anatomy characteristics. Some patients also do not respond well to radiotherapy and chemotherapy, and consequently, develop rapid recurrence after standard therapy (Tan et al., 2020). Despite recent progress in the development of new drugs, researchers are facing challenges in developing therapeutics for gliomas due to biological properties, including the blood-brain barrier, tumor specificity of gliomas, and immune environments (Aldape et al., 2019).

The tumor microenvironment (TME), as the “soil” for tumor survival, is crucial for tumor survival and is closely associated with the malignant behavior of tumor cells equivalent to “seed.” Compelling evidence shows that tumor cells interact with the extracellular matrix (ECM), immune cells, chemokines, and cytokines to create a favorable microenvironment for the proliferation and metastasis of tumors (Maman and Witz, 2018). The microenvironment of different tumors is diverse. For instance, in glioma, tumors can cooperate with peritumoral cells through chemokines and cytokines, direct contact, extracellular vesicles, nanotubes and microtubules, to promote tumor proliferation, brain invasion, angiogenesis and immunosuppression. This consequently creates a microenvironment conducive to the growth of malignant tumors (Broekman et al., 2018). Therefore, an in-depth understanding of the interactions between tumor cells and peritumoral cells may provide a new perspective to managing gliomas.

Interestingly, cancer-associated fibroblasts (CAFs) are an important component of stromal cells in TME and an “architect” of matrix remodeling, which are closely related to the prognosis of solid tumors (Cox, 2021). In TME, CAFs play a key role in the induction of Epithelial-mesenchymal Transition (EMT), and the maintenance of the pool of cancer stem cells and drug resistance by interacting with tumor cells and immune cells and releasing a variety of soluble factors (Su et al., 2018; Erin et al., 2020). Moreover, CAFs regulate tumor immunity and promote immune escape and resistance to cancer immunotherapy (Liu et al., 2019a). Therefore, targeting CAFs would not only inhibit the “seed” of cancer but also transform the “soil” into a microenvironment that inhibits tumor growth, and consequently transform the “enemy” that promotes tumor progression into a “friend” that inhibits tumor growth or metastasis (Chen and Song, 2019). Also, CAFs are activated to different degrees at different stages of tumor development. Studies have shown that different cytokines secreted by CAFs can play a pro-cancer or anti-cancer role (Liu et al., 2019b; Wang et al., 2021).

It is also noteworthy that because glioma is a “cold” tumor, immunotherapy has poor efficacy against the malignancy (Jackson et al., 2019). As such, to improve the efficacy of immunotherapy, transforming the “cold” environment into a “hot” one without causing neurotoxicity is imperative. Additionally, novel immunotherapeutic approaches, including oncolytic virus and adoptive T cell therapy, may exploit the T cell response to overcome the “cold” state of glioma, such as GBM (Buerki et al., 2018). Intriguingly, CAFs are co-expressed with the Fibroblast Activation Protein α (FAPα) and the Platelet-derived Growth Factor Receptor (PDGFR), which are the main components of stromal cells in GBM. Oncolytic adenoviruses have also been shown to specifically target GBM cells and CAFs (Li et al., 2020).

Cancer-associated fibroblasts are highly heterogeneous in terms of tissue origin, phenotype and function (Louault et al., 2020), but their function in the glioma microenvironment is yet to be fully elucidated. In the present study, we analyzed the sequencing data of glioma cohorts from the Chinese Glioma Genome Atlas (CGGA) database by the Estimate Proportion of Immune and Cancer cells (EPIC) algorithm (Racle et al., 2017; Liu et al., 2020), which is a common method to analyze the cell types based on the gene expression profile of the different type of cells, to quantify the expression of CAFs, then further analyze the relationship among CAFs and clinical features, tumor purity, immune score, stromal score, ESTIMATE score and stemness score of gliomas. Differentially expressed genes related to prognosis in the high and low expression subtypes of CAFs were also screened. Moreover, the risk scores of gliomas were assessed according to the expression of six genes identified as independent prognostic factors via the Least Absolute Shrinkage and Selection Operator (LASSO) regression analysis and validated in the Cancer Genome Atlas (TCGA) database. The established risk signature containing six CAFs-related-genes strongly correlates with the clinical and immune characteristics of glioma, including immune cells, immune checkpoints, and immunotherapy.

## Materials and Methods

### Data Collection

The RNA sequencing datasets [mRNAseq_693, mRNAseq_325 and mRNA sequencing (non-glioma as a control)] and the corresponding clinical and molecular information, including sex, age, grade, IDH status, 1p/19q status, MGMT promoter, and survival status information, were retrieved from the CGGA database^1^ (Zhao et al., 2021). The data were categorized into the training cohort [including 625 cases of low-grade gliomas (LGG) and 388 cases of GBM] and the testing cohort (comprising transcriptome data from 698 cases of gliomas in TCGA database from the Genomic Data Commons Data Portal (GDC).^2^ Additionally, the FPKM data was converted into TPM for subsequent analysis. The mRNAseq_693 and mRNAseq_325 data were merged into one metadata set, and batch effects were removed using the combat function in the SVA R package.

### Quantification of Different Cell Types in the Tumor Microenvironment

Estimate Proportion of Immune and Cancer cells (Racle et al., 2017) is an effective algorithm used to simultaneously estimate the proportion of cancer and immune cell types according to the gene expression in tumors based on a unique set of RNA-seq reference gene expression profiles described previously (Tirosh et al., 2016). It allows for accurate prediction of the proportion of cancer and non-malignant cell types even in the absence of *a priori* information about cancer cells. In this study, the reference profiles from tumor-infiltrating cells were used as the parameter, whereas the TPM data of CGGA glioma were used as input data. The proportion of CAF was estimated using the R package “EPIC” to explore the changes of matrix components in gliomas. CIBERSORT is a tool that deconvolutes the expression matrix of human immune cell subtypes based on the principle of linear support vector regression. Researchers also apply this tool to determine immune cell composition, which comprises 22 immune cell subtypes, based on the specific gene expression data of the cells (Newman et al., 2015). We analyzed gene expression data with standard annotation using CIBERSORT source code in relative mode. The algorithm was run using the LM22 signature and 100 permutations. For each sample, the final CIBERSORT output estimates were normalized to sum to one such that it could be interpreted directly as cell fractions for comparison across different immune cell types and datasets. Immune infiltration analysis based on a single-sample gene set enrichment analysis (ssGSEA) score can also be employed to explore the degree of immune infiltration of gliomas. It defines an enrichment score representing the absolute degree of enrichment of the gene set in each sample in each dataset. ssGSEA-based evaluation of the level of immune infiltration in a sample according to the expression levels of immune cell-specific marker genes demonstrated the immune infiltration landscape of gliomas (Bindea et al., 2013). The ESTIMATE package was used to estimate tumor immune score and tumor purity (Yoshihara et al., 2013). The stemness index of glioma was calculated according to the expression of tumor stem cell genes (Miranda et al., 2019). Submap^3^ algorithms (Hoshida et al., 2007) were further utilized to predict the clinical response to immune checkpoint blocking therapy for PD-1 and CTLA4 in the low-and high-risk score groups. *P*-value < 0.05 denoted statistical significance.

### Identification of Risk Genes and Calculation of Risk Score

Data were grouped into high subtype and low subtype according to the median value of CAFs. This was followed by the analysis of the differences between the mRNAseq_325 and mRNAseq_693 CGGA data. Differentially expressed genes (DEGs) were obtained according to a fold change (logFC) > 1.5 and *P* < 0.05. Genes with *P* < 0.001 and genes associated with prognosis were screened through univariate Cox regression analysis. A total of 329 genes were identified from mRNAseq_325 and 116 from the mRNAseq_693 CGGA database. Crossing the two sets of genes yielded 104 genes. Furthermore, multivariate Cox regression analysis was performed to identify 11 genes related to glioma overall survival (OS) (*P* < 0.001). Subsequently, LASSO analysis was employed, to minimize the risk of overfitting a prognostic model and construct a risk model (Tibshirani, 1997). The LASSO was utilized for variable selection and shrinkage via the “glmnet” package in R software (Simon et al., 2011). The independent variable in the regression was the normalized expression matrix of candidates for prognostic factors. The response variable was the OS time and state of the patients in the CGGA cohort. The penalty parameter (λ) of the model was constructed through tenfold cross-validations, followed by the minimum criteria (i.e., the value of λ corresponding to the lowest partial likelihood deviance). The risk score of the patients was calculated according to the normalized expression level of the prognostic gene signature and their corresponding regression coefficients according to the formula: risk score = e^sum (each gene^′^s^ ^expression × corresponding coefficient)^. Finally, patients were divided into high and low risk groups according to the median risk score.

### Establishment of the Nomogram

The nomogram incorporated age, grade, 1p/19q status based on the CGGA cohort. The prognostic risk score model was established via the “RMS” package in R. The consistency between predicted survival rate and actual survival rate using time-dependent calibration curves, and verified in the TCGA cohort. The concordance index (C index) was calculated to evaluate the effectiveness of the model in prognosis prediction. The C index ranged between 0.5 and 1.0; notably, a higher index denoted the better the performance of the model in predicting survival rate.

### Functional Enrichment Analysis

Spearman’s correlation analysis was used to reveal the genes associated with the risk score; here, the correlation coefficient ≥ 0.4 was selected. Genes related to risk scores were sorted according to the calculated correlation coefficient. Next, Gene Set Enrichment Analysis (GSEA) was performed with the “clusterProfiler” R package (Yu et al., 2012), using “h.all.v7.0.entrez.gmt” as a reference gene set. *P* values were adjusted using the Benjamini and Hochberg methods. *P*-value < 0.05 implied statistically significant differences. Lastly, the results of the first five enrichment analysis were visualized using “enrichplot” and “ggplot2” (Wickham, 2016) R software package.

### Statistical Analysis

Patients in the CGGA training and TCGA validation cohorts were categorized into the high- and low-risk subtypes according to the median risk score. Wilcoxon rank-sum test was applied to compare the high- and low-risk subtypes. Differences among three or more subtypes of patients were tested using the K-W test. Kaplan-Meier analysis and log-rank test were employed to analyze survival rates between low-risk and high-risk subtypes. Univariate and multivariate Cox regression analyses identified the independent factors associated with the OS of glioma. All statistical analyses were conducted in R software (version 4.0.3), and *P* < 0.05 denoted statistical significance (^∗^*P* < 0.05, ^∗∗^*P* < 0.01, ^∗∗∗^*P* < 0.001, ^****^P < 0.0001).

## Results

### Cancer-Associated Fibroblasts Are Closely Related to the Clinical Progression of Gliomas

**FIGURE 1:**
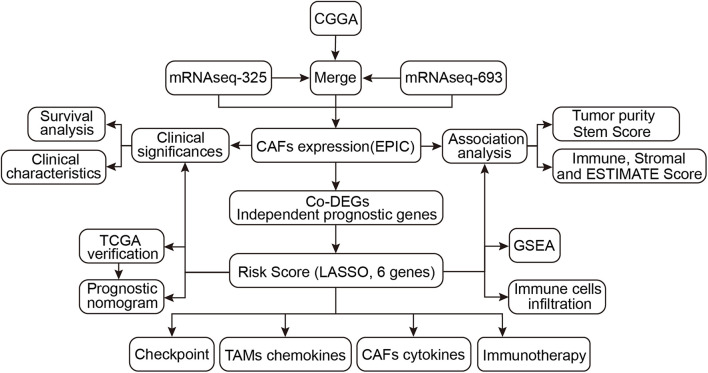
Flowchart of the study.

**FIGURE 2:**
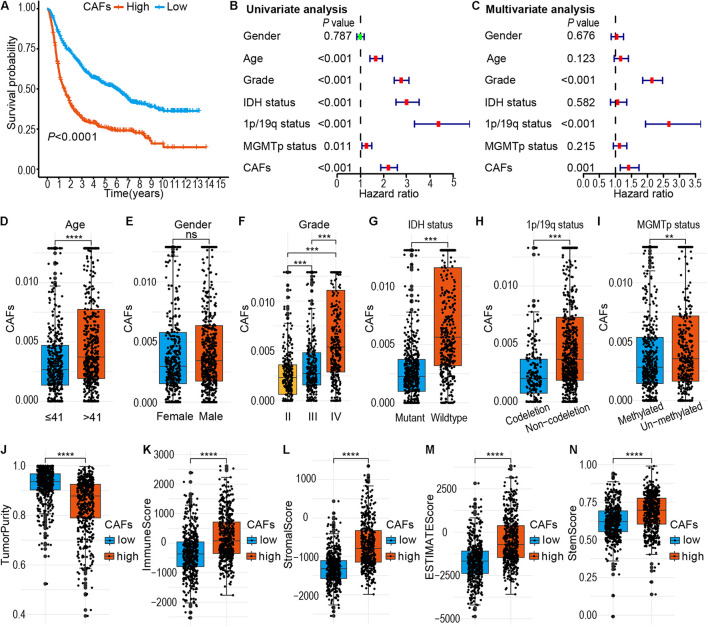
Predictive values of cancer-associated fibroblasts (CAFs) in the survival of glioma patients and its relationship with pathological characteristics. **(A)** Comparison of overall survival between low and high CAFs subtypes in the Chinese Glioma Genome Atlas (CGGA) database. **(B)** Univariate Cox regression analysis. Forest plot of the association between CAFs and glioma survival. **(C)** Multivariate Cox regression analysis showed that the CAFs was an independent predictor of gliomas. **(D–N)** Distribution of the CAFs in patients stratified according to age **(D)**, gender **(E)**, WHO grade **(F)**, isocitrate dehydrogenase (IDH) status **(G)**, 1p/19q status **(H)**, O-6-methylguanine-DNA methyltransferase (MGMT) promoter status **(I)**, Tumor purity **(J)**, Immune score **(K)**, Stromal score **(L)**, ESTIMATE score **(M)**, and Stemness score **(N)**. ****P < 0.0001; ***P < 0.001; **P < 0.01; ns, no significant.

A detailed flow chart of this analysis is shown in Figure 1. Using EPIC, the proportion of CAFs was calculated in different glioma samples in the CGGA cohort. The mRNAseq_693 and mRNAseq_325 datasets in the CGGA cohort were merged and batch discrepancies were eliminated. Subsequently, the proportion of CAFs was calculated in gliomas in the training cohort. Patients were then stratified into high and low CAFs subtypes according to the median of CAFs. The prognosis of the high-CAFs subtype was worse than the low-CAFs subtype (log-rank, *P* < 0.0001; Figure 2A). According to the WHO (2016) grading guidelines, patients with high CAFs showed poor prognosis in Oligodendroglioma with IDH mutation and 1p/19q co-deletion, Astrocytoma with IDH mutant, Astrocytoma with IDH wildtype, GBM with IDH mutant, and GBM with IDH wildtype (Supplementary Figure 1). In addition, both univariate and multivariate Cox regression analyses verified that the proportion of CAFs is an independent risk predictor for gliomas (Figures 2B,C). On the other hand, the high proportion of CAF is mainly enriched in glioma patients with higher age (*P* < 0.0001), high WHO grade (*P* < 0.001), IDH wildtype status (*P* < 0.001), 1p/19q non-codeletion status (*P* < 0.001), and MGMT promoter un-methylated status (*P* < 0.01). However, CAFs did not differ between genders (Figures 2D–I). To eliminate the effect of data consolidation in mRNAseq_693 and mRNAseq_325 datasets, the proportion of CAFs in both datasets was calculated, respectively, which yielded a similar result to the merged dataset (Supplementary Figure 2). Results suggest that the proportion of CAFs is of promise as an independent predictor for the prognosis and progression of gliomas.

### A High Proportion of Cancer-Associated Fibroblasts Is Associated With Immune Landscape and Stemness of Gliomas

Cancer-associated fibroblasts as the architect of matrix remodeling in TME may affect cell components of TME and promote progression of tumor malignancy. In this study, the ESTIMATE and GSVA packages in R were used to estimate tumor purity, immune score, stromal score, ESTIMATE score, and stemness score of gliomas in the CGGA cohort. A high proportion of CAFs was revealed to be associated with the glioma patients with low tumor purity, high stromal score, high ESTIMATE score, and high stemness score (*P* < 0.0001; Figures 2J–N). At the same time, the prognosis of glioma patients characterized by low tumor purity, high immune score, high stromal score, high ESTIMATE score, and high stemness score was poor (*P* < 0.0001, Supplementary Figures 3A–E). The findings suggest the association of low tumor purity, high immune score, high stromal score, high ESTIMATE score, and high stemness score with a high proportion of CAFs and the OS of glioma patients.

### Construction of Prognostic Gene Signatures of Cancer-Associated Fibroblasts Which Are Related to the Status Gliomas

**FIGURE 3:**
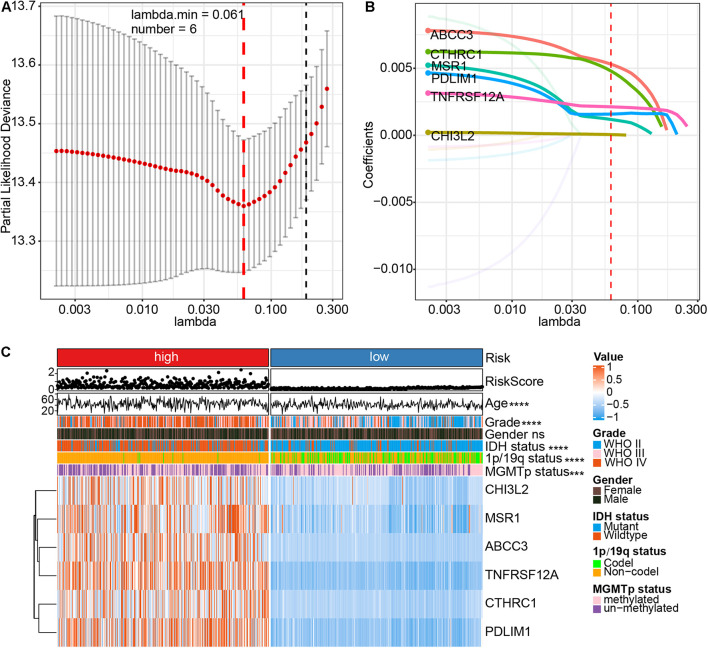
Identification of a CAFs-related six-gene risk signature for overall survival risk characteristics by least absolute shrinkage and selection operator (LASSO) regression analysis in CGGA cohort. **(A)** Cross-validation for tuning parameter (lambda) screening in the LASSO regression model. **(B)** LASSO coefficient spectrum of six genes in gliomas. **(C)** The heatmap shows the association between risk and clinic pathological characteristics of the six-gene risk signature. LASSO, least absolute shrinkage and selection operator. ****P < 0.0001; ***P < 0.001; ns, no significant.

After calculating the proportion of CAFs in the CGGA mRNAseq_693 and mRNAseq_325 cohorts, the training cohorts were stratified into high and low subtypes according to the median of CAFs. Subsequently, univariate Cox regression analysis was conducted on both datasets of DEGs to screen for genes related to prognosis (with *P* < 0.001). Through a crossover analysis of the two DEGs sets, 104 genes were obtained. Eleven genes related to the total survival of glioma patients were obtained via multivariate Cox regression analysis (*P* < 0.001). The risk of overfitting was minimized using the LASSO regression algorithm. The risk score was calculated according to the expression level and regression coefficient of six genes (ABCC3, CTHRC1, MSR1, PDLIM1, TNFRSF12A, and CHI3L2) (Figures 3A,B). The risk score = (ABCC3 × 0.00530703547540297) + (PDLIM1 × 0.0015 8225241997429) + (CHI3L2 × 0.0000527762285183538) + (MSR1 × 0.00118065457455651) + (CTHRC1 × 0.004799633 48819953) + (TNFRSF12A × 0.00212454022154963). The formula was also used to calculated risk scores for the glioma patients in the TCGA validation cohorts. Of note, the six genes associated with the risk of glioma were highly expressed in the high-risk group (Figure 3C). There were significant differences in risk scores among patients with different ages in terms of first diagnosis (*P* < 0.001), WHO grade (*P* < 0.0001), IDH (*P* < 0.0001), 1p/19q (*P* < 0.0001) and MGMT promoter (*P* < 0.001). These six genes were also mainly highly expressed in high-grade glioma and IDH wildtype groups (Supplementary Figures 4A–L). These results strongly demonstrated that the risk model, based on the six genes is closely associated with the clinical progression of glioma, therefore, could be employed as a risk prediction model for glioma.

### Survival Analysis and Pathological Features Between High- and Low-Risk Patients

**FIGURE 4:**
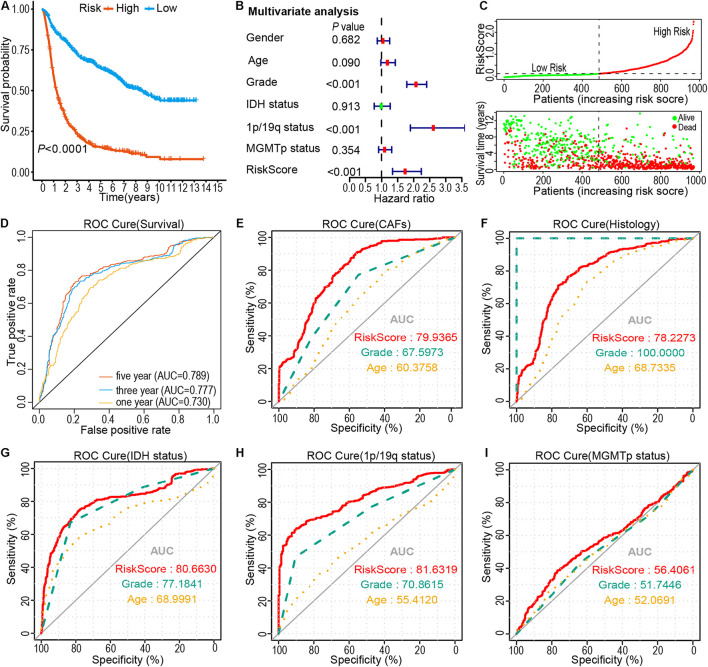
Prognostic significance of risk signature derived risk score in the CGGA cohort. **(A)** Kaplan-Meier analysis of CGGA glioma patients was stratified by median risk. **(B)** Multivariate Cox regression analysis showed that the risk signature was an independent predictor of gliomas. **(C)** Risk score distribution, patient survival time, and glioma status. The black dotted line is the optimal threshold for classifying patients into low-and high-risk subtypes. **(D–I)** A high-risk score is associated with a lower survival rate for gliomas. ROC curves showed the predictive efficiency of risk characteristics, overall survival **(D)**, CAFs **(E)**, Histology **(F)**, IDH status **(G)**, 1p/19q status **(H)**, and MGMT promoter methylation status **(I)**.

Kaplan-Meier analysis showed that the OS of the high-risk subtype was worse compared to that of the low-risk subtype (*P* < 0.0001; Figure 4A). Additionally, the risk score was related to a prognostic value in gliomas, stratified according to the WHO guidelines (2016) for the grading of tumors. The high-risk subtype was related to the low OS of the patients with Oligodendroglioma with IDH mutant and 1p/19q co-deletion, Astrocytoma with IDH mutant, Astrocytoma with IDH wildtype, GBM with IDH mutant, and GBM with IDH wildtype (Supplementary Figure 5). Next, we performed a multivariate Cox regression analysis of the risk score and clinical-pathological features of glioma patients. Results demonstrated that risk score is an independent risk factor to predict the OS in patients with glioma (HR = 1.731, 95% CI = 1.341–2.237, *P* < 0.001; Figure 4B). The heatmap showed that high glioma mortality is related to an increased risk score (Figure 4C). Furthermore, through ROC curve analysis, the accuracy of risk score as a prognostic factor for glioma was validated. Results showed that the risk score could predict the OS of the CGGA cohorts (5-year, AUC = 0.789). Of note, higher CAFs (AUC = 0.7994), tumor histology (AUC = 0.7822), IDH status (AUC = 0.8066), 1p/19q status (AUC = 0.8163) and MGMT promotor status (AUC = 0.5641) was shown in the high-risk group than in the lower group (Figures 4D–I). Moreover, glioma data from TCGA was utilized to validate the risk score. The LASSO regression analysis was performed on the TCGA data to calculate the patients’ risk scores using similar regression coefficients. Subsequently, K-M survival analysis of the TCGA data was performed to assess the risk model. Results demonstrated lower OS in the high-risk subtype than that in the low-risk subtype (Supplementary Figure 6A). Univariate Cox regression analysis was conducted to explore the prognostic value of the risk score. Results showed a significant correlation of the risk score with OS in TCGA LGG-GBM (HR = 4.255, 95% CI = 3.538–5.117, *P* < 0.001, Supplementary Figure 6B). Moreover, multivariate Cox regression analysis demonstrated that the risk score was an independent prognostic indicator (HR = 1.888, 95% CI = 1.438–2.4, *P* < 0.001, Supplementary Figure 6C). ROC curve analysis revealed that the risk model had a strong predictive value for the OS of glioma patients (Supplementary Figure 6D). Furthermore, six CAFs-related genes were highly expressed in TCGA LGG-GBM cohorts, particularly in the high-risk subtype (Supplementary Figure 6E), consistent with the results of CGGA cohorts. Taken together, the results provided evidence that the high-risk subtype is associated with low OS rate and some clinical-pathological features, therefore, has application prospects as a risk model based on the specific expression of the six genes.

### The Risk Model for Individual Prognostic Prediction

**FIGURE 5:**
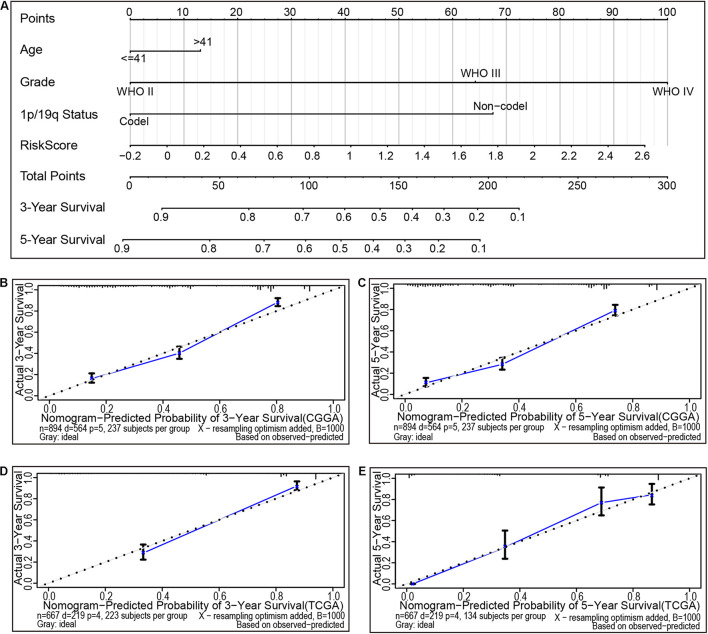
The nomogram can predict the prognosis of gliomas. **(A)** A nomogram of the gliomas cohort (training set) was used to predict overall survival. **(B,C)** A calibration plot was used to predict the 3-year **(B)** and 5-year survival **(C)** in the CGGA cohorts (training set). Calibration plots for 3-year **(D)** and 5-year survival **(E)** in the Cancer Genome Atlas (TCGA) cohort (testing set). The X- and Y-axis represent the Nomogram prediction and actual survival, respectively. The solid line represents the nomogram of the forecast and the vertical line represents the 95% confidence interval.

Nomogram is a powerful tool used to quantitatively determine individual risk in the clinical setting by integrating various risk factors. Herein, using a six CAFs-related genes signature, a nomogram was constructed based on age, grade, 1p/19q gene deletion status, and risk score to predict the probability of 3- and 5-year OS. Meanwhile, the calculated C index was 0.760. Each factor was graded according to its contribution to OS (Figure 5A). The calibration curve showed a consistent actual survival rate with the predicted survival rate (Figures 5B,C). The accuracy of the prognostic prediction model was verified in the TCGA cohort, and the calculated C index was 0.844. The correction chart demonstrated that the 3- and 5-year OS corroborated with the predicted values (Figures 5D,E). These data demonstrate that the risk model is relatively accurate and can improve the ability to estimate individual prognosis.

### The Immune Landscape Between Different Risk Subtypes

**FIGURE 6:**
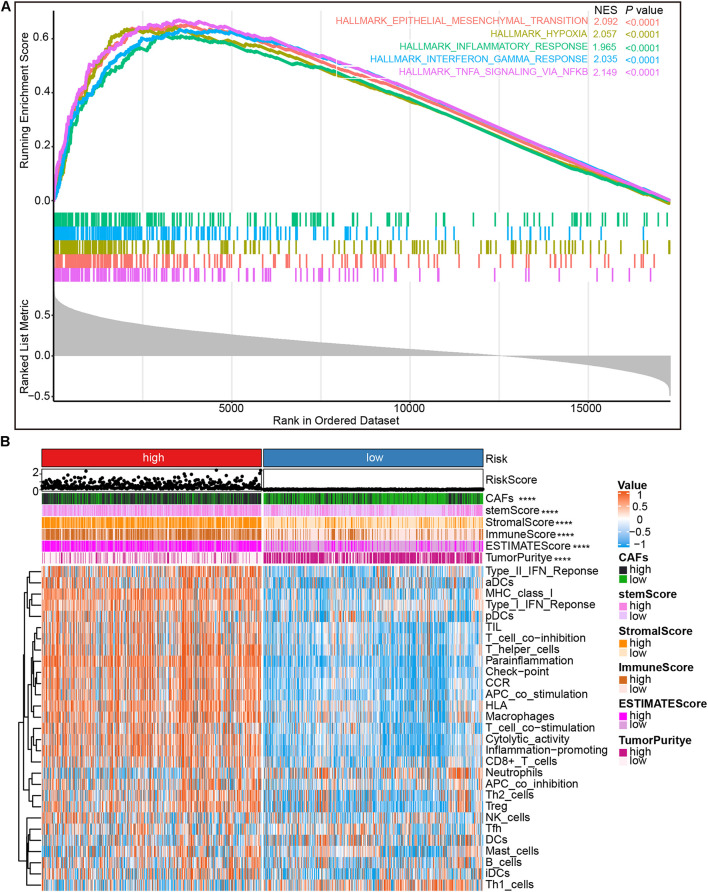
Gene set enrichment analysis (GSEA) and immune cell infiltration analysis. **(A)** The GSEA of genes positively correlated with risk score. **(B)** Heatmap showing immune cells infiltration by ssGSEA algorithm between low- and high-risk groups in the CGGA cohort. NES, normalized enrichment score. ****P < 0.0001.

To determine the potential biological processes of risk score, Spearman’s correlation analysis was performed to identify the genes related to the risk score. Genes with Spearman correlation coefficient ≥ 0.4 were used for GSEA analysis. Results revealed that genes associated with risk score were mainly associated with epithelial-mesenchymal transformation (*P* < 0.0001, *q* < 0.0001), hypoxia (*P* < 0.0001, *q* < 0.0001), inflammation (*P* < 0.0001, *q* < 0.0001), interferon-gamma response (*P* < 0.0001, *q* < 0.0001), and NFkB-mediated TNFα signal transduction (*P* < 0.0001, *q* < 0.0001; Figure 6A). Furthermore, the ESTIMATE and GSVA packages in R were employed to estimate tumor purity, immune score, stromal score, ESTIMATE score, stemness score, and the proportion of immune cells in the CGGA cohort. Results showed that the high-risk subtype was associated with a high proportion of CAFs, high stemness score, high stromal score, high immune score, the high ESTIMATE score, and low tumor purity (*P* < 0.0001; Figure 6B). In addition, aDC, pDC, iDC, T helper cells, macrophage, Th2 cells, Treg cells, and B cells were rich in high-risk subtype, whereas neutrophil, Th1 cells were rich in low-risk subtypes (Figure 6B). To further verify that immunosuppressive cells were mainly enriched in the high-risk group, a histogram was generated and the proportion of immune cells in the CGGA data was calculated via the CIBERSORT algorithm. Results revealed that immunosuppressive cells, including Tregs and M2 macrophages, were mainly concentrated in the high-risk subtype (Supplementary Figures 7A,B). Intriguingly, the proportion of immune cells calculated by ssGSEA and the CIBERSORT algorithm showed that CD4^+^, CD8^+^ T cells, and NK cells were also enriched in the high-risk subtype (Supplementary Figure 7C).

### The Immune Checkpoint Landscape Between Different Risk Subtypes

**FIGURE 7:**
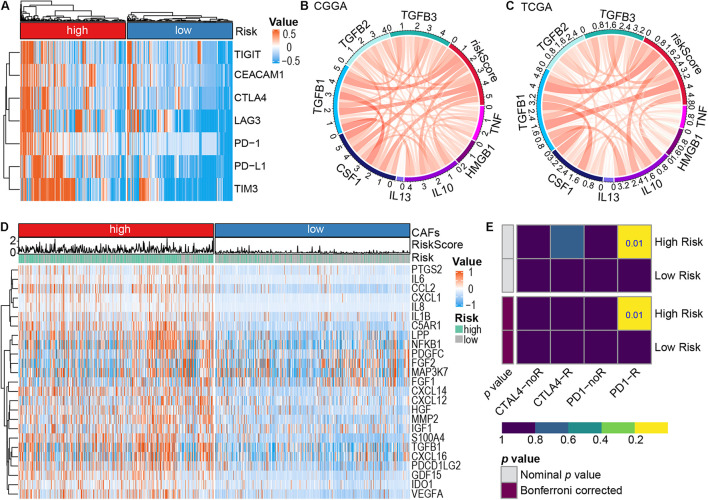
The role of risk signature in immune microenvironment and immunotherapy. **(A)** Expression of immune cell exhaustion marker genes in the high- and low-risk subtypes. **(B,C)** Correlation between representative macrophage chemokines and risk score, CGGA dataset **(B)**, TCGA dataset **(C)**. M1 macrophage chemokines (TNF, HMGB1), M2 macrophage chemokines (IL10, IL13, CSF1, TGFB1, TGFB2, and TGFB3). **(D)** Cytokines secreted by CAFs high expression in the high-risk subtype. **(E)** Predicting response to immunotherapy (anti-PD-1 and anti-CTLA4) in the high-and low-risk subtype based on the Submap algorithms.

Previously, T cells and NK cells exhaustion have been demonstrated to potentially facilitate cancer cells to escape host immunity, which is linked to poor prognosis (Gonzalez-Gugel et al., 2016; Zarour, 2016). In the present work, most of these exhaustion markers [TIGIT (Manieri et al., 2017), CEACAM1 (Huang et al., 2015), CTLA4 (Krummel and Allison, 1996; Postow et al., 2015), LAG3 (Ruffo et al., 2019), PD-1 (Ishida et al., 1992; Postow et al., 2015), PD-L1 (Postow et al., 2015), and TIM3 (Huang et al., 2015)] were highly expressed in the high-risk subtype (Figure 7A), indicating an elevated level of immune exhaustion in the tumors of high-risk glioma patients. It is well known that CAFs can promote the transformation of M1 macrophages to M2 and induce the secretion of related cytokines to promote tumor invasion, angiogenesis, and change the immune landscape of tumors (Farhood et al., 2019; An et al., 2020). Therefore, we explored the expression of chemokines and cytokines secreted by CAFs in the high-risk subtype and the low-risk subtype. M2 macrophage chemokines (IL10, IL13, CSF1, TGFB1, TGFB2, and TGFB3) were found to be highly positively correlated, whereas M1 macrophage chemokines (HMGB1, TNF) were weakly positively correlated with risk score (Zhang et al., 2021; Figures 7B,C). Additionally, analysis of the expression of chemokines and cytokines secreted by CAFs in the high-risk subtype and the low-risk subtype demonstrated that most of the cytokines secreted by CAFs were highly expressed in the high-risk subtype (Figure 7D). Patients with a high-risk score exhibited a type I interferon response, type II interferon response, and the activation of pro-inflammatory functions (Supplementary Figure 7C). These observations provided evidence that patients with a high-risk score are eligible for and respond to immunotherapy. Furthermore, subclass mapping was applied to compare the expression profile of the high- and low-risk subtypes with another published dataset containing 47 patients with melanoma that responded to immunotherapies (Roh et al., 2017). The high-risk subtype showed potent responses to anti–PD-1 therapy (Bonferroni correction *P* = 0.01; Figure 7E).

## Discussion

The present study, through analysis of the CGGA and TCGA databases of glioma, revealed that CAFs are associated with clinical and immunological characteristics of gliomas. More evidence shows that CAFs are an independent prognostic factor and CAFs are enriched in patients with a poor survival rate, older age, high tumor grade, IDH wild-type, 1p/19q non-codeletion, and MGMT promoter unmethylation (Figures 2A-I). This work has also demonstrated the association of a high proportion of CAFs in glioma patients with low tumor purity and high immune score, high stromal score, ESTIMATE score, and high stemness score (Figures 2J-N). By intersecting the two DEGs sets and conducting a further screen through multivariate Cox regression analysis, the remaining prognostic genes were used for LASSO regression analysis. Assessment of the risk score was based on the expression of six CAFs-related genes (ABCC3, CTHRC1, MSR1, PDLIM1, TNFRSF12A, and CHI3L2) (Figure 3). Similar to the proportion of CAFs, the risk score was correlated with a survival rate, clinical characteristics, tumor purity, immune score, stromal score, ESTIMATE score, and stemness score. These data strongly demonstrate that the risk score holds great promise as an independent prognostic factor for glioma (Figure 4). Furthermore, independent prognostic factors were used to construct a nomogram to directly predict individual OS based on six CAFs-related-gene signature risk score (Figure 5). It was revealed that the main enriched pathways in the tumor, including epithelial-mesenchymal transformation (Shintani et al., 2016), hypoxia (Lappano et al., 2020), inflammatory Response (Ershaid et al., 2019), interferon-gamma response (Broad et al., 2021), and NFkB-mediated TNFα signal transduction (Katanov et al., 2015) were closely related to the function of CAFs. These observations indicate that risk scores can fully reflect the function of CAFs (Figure 6A). To explore the role of the risk score in the TME, ssGSEA, and CIBERSORT were employed to evaluate immune cell infiltration. Percentage analysis of immune cells demonstrated that gliomas were mainly enriched with macrophages and few T cells. Also, significant differences in the abundance of immune cells between the high-and low-risk subtypes were noted. Th1 cells were mainly enriched in the low-risk subtype while immunosuppressive cells such as Th2 cells, Tregs, and M2 macrophages were mainly enriched in the high-risk category. Furthermore, CD4^+^ T cells, CD8^+^ T cells, and NK cells were highly enriched in the high-risk subtype (Figure 6B and Supplementary Figure 7). In addition, high-risk patients were shown to express high levels of markers of immune cell exhaustion (Figure 7A). These data demonstrate that although immune-activated cells are highly expressed in the high-risk group, it is possible that they are in a state of functional inhibition.

High expression of immune-related cytokines and chemokines in high-risk subtypes provide evidence on the relationship between CAFs and tumor-associated macrophages (TAMs) (Figures 7B–D). Studies show that recruitment of TAMs into the glioma environment may induce immunosuppression and tumor promotion effects (Hambardzumyan et al., 2016). Moreover, CAFs may promote the polarization of macrophages to M2 macrophages and exert an immunosuppressive effect in TME, and more, CAFs can secret cytokines and chemokines to interact with various immune cells in the tumor environment, contributing to the malignant transformation of tumors and resistance to treatment (Linares et al., 2020). Therefore, secretion factors from CAFs are promising indicators for tumor diagnosis and prognosis, and as drug targets.

Previous evidence indicates that CCL2 in the glioma microenvironment promotes the recruitment of Tregs and myeloid-derived suppressor cells (MDSCs) (Chang et al., 2016). On the other hand, CD70^+^ CAFs are independent markers for poor prognosis in invasive colorectal cancer, they potentially increase the infiltration of Tregs and promote the immune escape of tumor cells (Jacobs et al., 2018). Immunosuppression mediated by high expression of PD-L1 in gliomas has been demonstrated to be potentially associated with the infiltration of TAMs and M2 polarization (Zhu et al., 2020). Studies have also shown that the differentiation and survival of macrophages are dependent on the colony-stimulating Factor-1 (CSF-1), and IL6 and the granulocyte-macrophage colony-stimulating factor (GM-CSF) secreted by CAFs, which promote the differentiation of monocytes into M2-like TAMs (Cho et al., 2018). Consistently, Pyonteck et al. (2013) found that targeting TAMs with the CSF-1R inhibitor significantly improves the survival rate of GBM mice. These pieces of evidence indicate that CAFs and their secreted factors orchestrate with the immune cells in the TME to promote glioma progression.

Increasing evidence has shown that T cell dysfunction contributes to tumor immune escape in patients with gliomas (Mirzaei et al., 2017; Woroniecka et al., 2018). In the presence of adenosine, activation of CD8^+^ T cells effectuates a decrease in the expression of IFN-γ and tumor necrosis factor-α (TNF-α), thereby inhibiting anti-tumor response (Takenaka et al., 2019). Moreover, CD8^+^ T cells during tumorigenesis mediate the immune editing of immunogenic tumor clones, contributing to immune escape in murine glioma (Kane et al., 2020). The present study, based on the six-gene signature of CAFs, reported a higher expression of immune checkpoint molecules in high-risk subtype tumors. It was notable that the functions of CAFs rely on immune checkpoint activation, which induces the loss of antigen specificity in CD8^+^ T cells and blocks the activity of T cells (Lakins et al., 2018). Furthermore, evidence indicates that to inhibit CD8^+^ T cells, CAFs can secrete high levels of IL-6, which remodel the immunosuppressive TME (Kato et al., 2018). Previous researchers also demonstrated that CAFs can recruit and balance CD4^+^ effector T cell subsets (Th1 and Th2), promoting the recruitment and differentiation of Tregs, and facilitate the transformation of macrophages to the M2 phenotype through frequent interactions with TAMs. Additionally, CAFs can inhibit the ability of CD8^+^ T cells to kill tumor cells. To achieve this, CAFs reduce T cell infiltration, blocking both the cytotoxic activity of T cells and T-cell communication in the TME (An et al., 2020; Baker et al., 2021). Taken together, these findings support the view that CAFs potentially inhibit CD8^+^ T function, promote the formation of MDSC, and establish an immunosuppressive TME to facilitate the immune escape of tumor cells.

Additionally, the present work revealed the activation of the inflammation-promoting function, type I IFN response, and type II IFN response in the high-risk subtype (Figure 6B and Supplementary Figure 7C). The expression levels of immune checkpoint-related genes were also higher in the high-risk subtype (Figure 7A), demonstrating that immunosuppressed patients in the high-risk category may respond to immune checkpoint blockers. By predicting the response and resistance of different risk subtypes to immune checkpoint blockers therapy, it was intriguing that patients with high-risk scores showed higher responses to anti-PD-1 therapy (Figure 7E). As such, patients in the high-risk subtype are likely to be more responsive to immunotherapy. These results demonstrate that the risk score based on the expression of the six CAFs-related genes is promising as a novel and reliable method for evaluating the prognosis and clinical response to immunotherapy of glioma patients.

More evidence shows that CAFs-induced inhibition of immunosuppression may further enhance the response of tumors to immunotherapy. Current evidence shows several therapeutic strategies that target CAFs, including (i) targeting cytokines and chemokines (such as TGF-β or IL6) through immunotherapy, directly consuming CAFs (such as FAP-DNA vaccine) via cell surface labeling; (ii) targeting CAFs through the elimination of endothelial progenitors using bevacizumab, normalizing activated CAFs (e.g., using VDR ligand calcipotriol); (iii) targeting CAFs-derived extracellular matrix proteins or their related signal transduction in animal models (Chen and Song, 2019; Liu et al., 2019a). Moreover, selectively targeting CAFs using nanomedicine has been revealed to enhance the infiltration of cytotoxic T cells and inhibits tumor growth (Zhen et al., 2017). More importantly, the synergistic effect of blocking immune checkpoint molecules and targeting CAFs can be achieved by remodeling the immunosuppressive microenvironment and achieving an immunotherapeutic response (Feig et al., 2013). Normalization of CAFs may eliminate the tumor-promoting effect and increase the sensitivity of treatment (Öhlund et al., 2014; Vennin et al., 2019). Collectively, CAFs secrete cytokines in the TME during glioma progression, interact with immune cells, mediate the formation of the immunosuppressive microenvironment and induce the transformation to the malignant phenotype. Therefore, an improved understanding of the interaction of CAFs with anti-tumor immunity is crucial in establishing effective immunotherapy. In this regard, approaches such as spatial transcriptomics, single-cell RNA sequencing (Moncada et al., 2020), and organoids (Xu et al., 2021) can be employed to comprehensively understand the Spatio-temporal dynamics of CAFs as they interact with tumor and immune cells and their role in TME of gliomas.

To the best of our knowledge, this is the first study to explore the clinical features of CAFs in gliomas and establish a prognostic signature, based on CAFs for predicting the survival outcome of glioma patients and immunotherapy efficacy. The results are based on public data sets analysis, therefore, more exploration is warranted on heterogeneity in different patient groups, including intertumoral or intratumoral heterogeneity. Also, the biological functions of six CAFs-related genes as a prognostic signature should be explored deeply. Notably, the roles of CAFs vary in different patients given the complex and diverse immune microenvironment of gliomas. Thus, the precise effects of CAFs and their interaction with tumor cells and immune cells deserve further clarification.

## Conclusion

Increased CAFs infiltration in gliomas is significantly correlated with older age, high tumor grade, IDH status, 1p/19q status and MGMT promoter status, tumor purity, immune score, stromal score, ESTIMATE score, stemness score, and patient prognosis. The established risk prediction model, based on the expression of six CAFs-related genes, has application prospects as an independent prognostic indicator. The risk model holds great promise in predict prognosis and immunotherapy response in glioma patients.

## Data Availability Statement

The datasets presented in this study can be found in online repositories. The names of the repository/repositories and accession number(s) can be found in the article/Supplementary Material.

## Author Contributions

HX and KY conceived and designed the study and drafted the manuscript. ZC, SZ, and GH performed data analysis and wrote the manuscript. JT, WH, and W-QG revised the manuscript. All authors reviewed the manuscript.

## Conflict of Interest

The authors declare that the research was conducted in the absence of any commercial or financial relationships that could be construed as a potential conflict of interest.

## Publisher’s Note

All claims expressed in this article are solely those of the authors and do not necessarily represent those of their affiliated organizations, or those of the publisher, the editors and the reviewers. Any product that may be evaluated in this article, or claim that may be made by its manufacturer, is not guaranteed or endorsed by the publisher.

## Footnotes

1
http://www.cgga.org.cn/

2
https://portal.gdc.cancer.gov/

3
https://cloud.genepattern.org/gp
