# Supplementary material for: Prognosis and Immunotherapy Significances of a Cancer-Associated Fibroblasts-Related Gene Signature in Gliomas
Source: Front Cell Dev Biol. 2021 Oct 29;9:721897. doi: 10.3389/fcell.2021.721897 (PMC8586504; doi:10.3389/fcell.2021.721897)
Supplement: Supplementary file 1 [file Data_Sheet_1.docx]

Supplementary Material

# Supplementary Figures


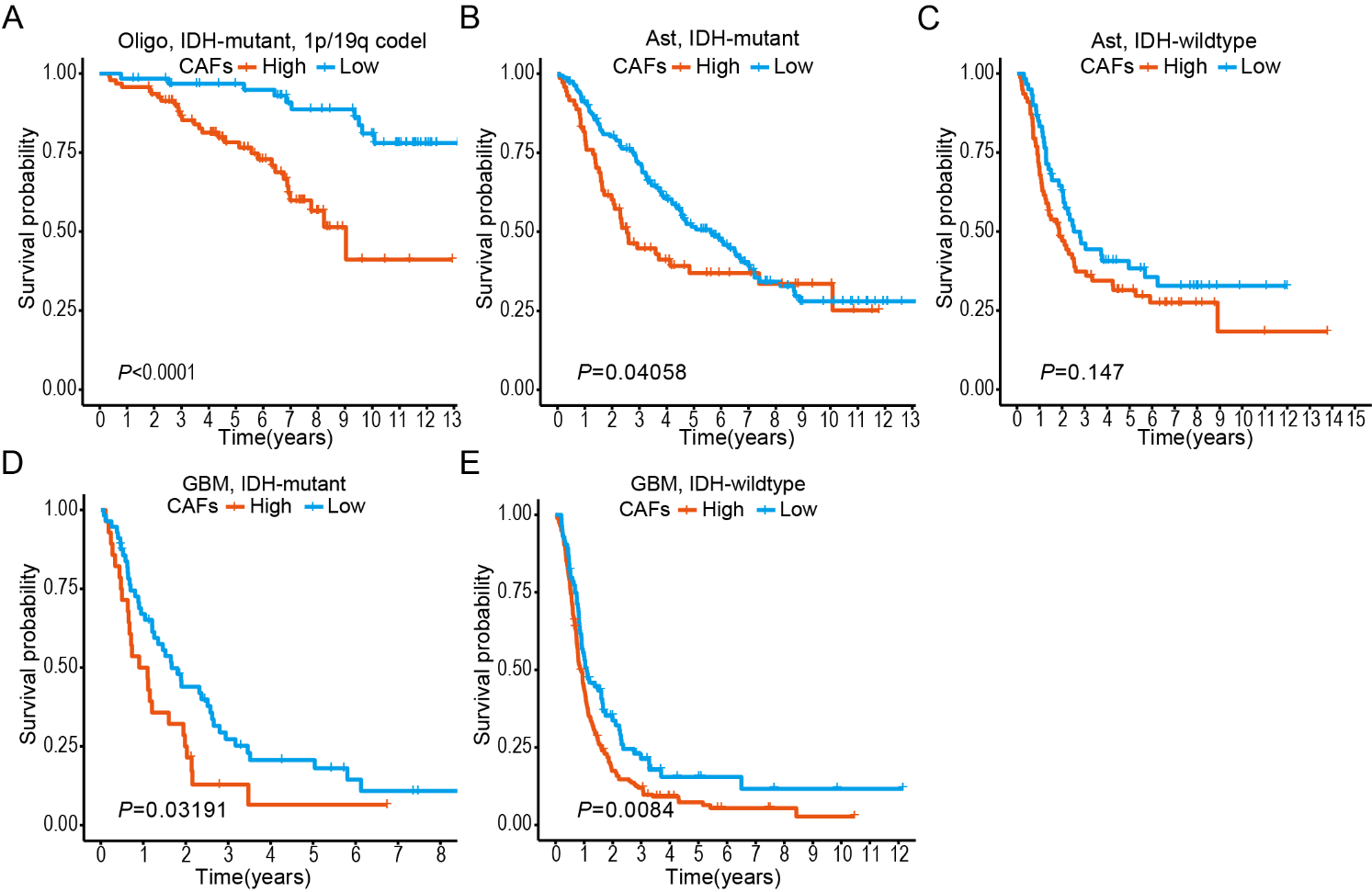


**Figure S1.** The prognostic value of stratified patients with CAFs was comprehensively analyzed by WHO 2016. (A-E) Kaplan–Meier overall survival curves in patients with Oligodendroglioma with IDH-mutant and 1p/19q co-deletion (A), Astrocytoma with IDH-mutant (B), Astrocytoma with IDH-wildtype (C), GBM with IDH-mutant (D), GBM with IDH- wildtype (E).


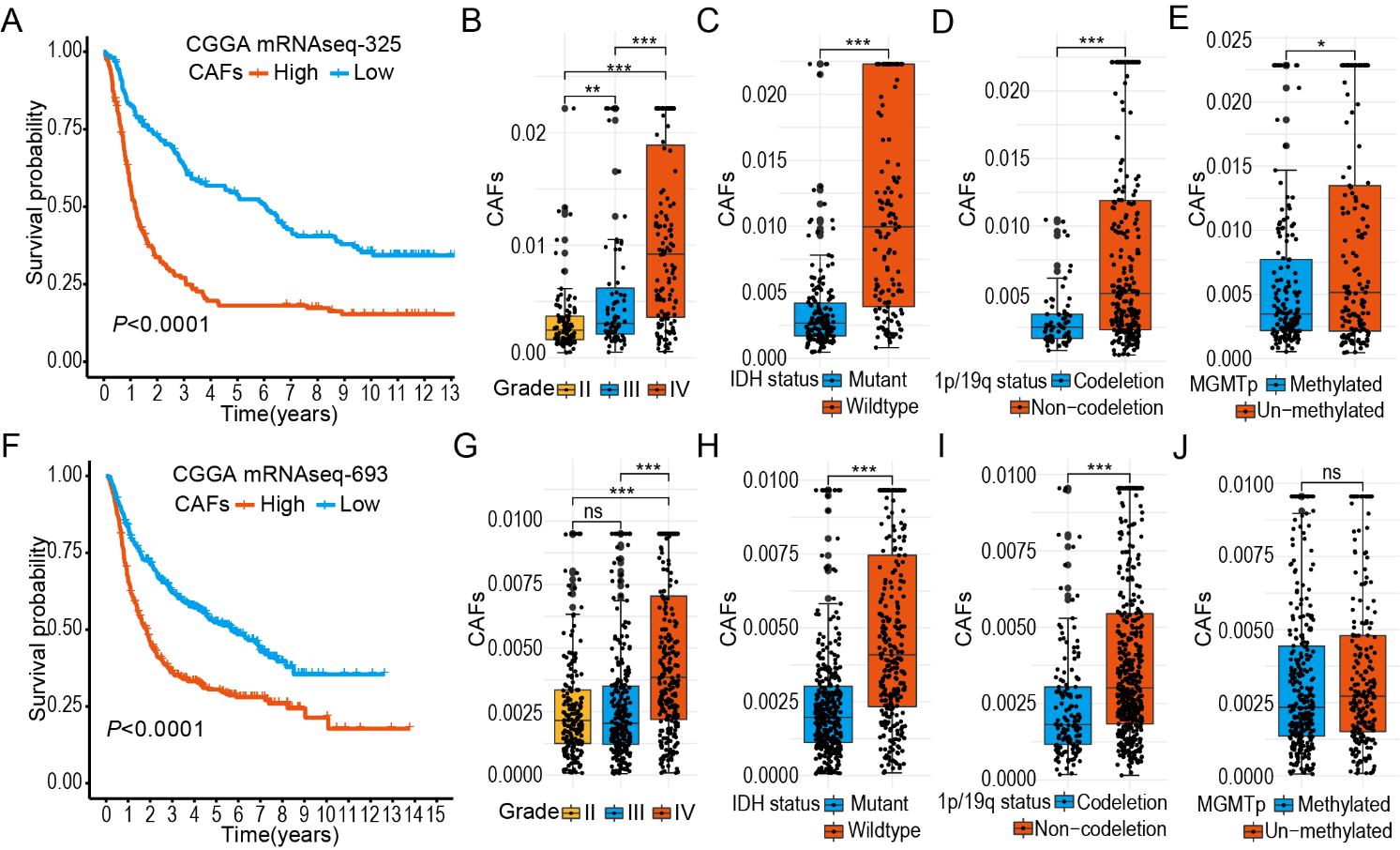
 **Figure S2.** The two sub-data of CGGA confirmed the predictive value of CAFs in the survival status of glioma patients and their relationship with pathological features. mRNAseq_325 (A-E), mRNAseq_693 (F-J). ***$\text{ P}$ < 0.001, **$\text{ P}$ < 0.01, *$\text{ P}$ < 0.05; ns, no significant.


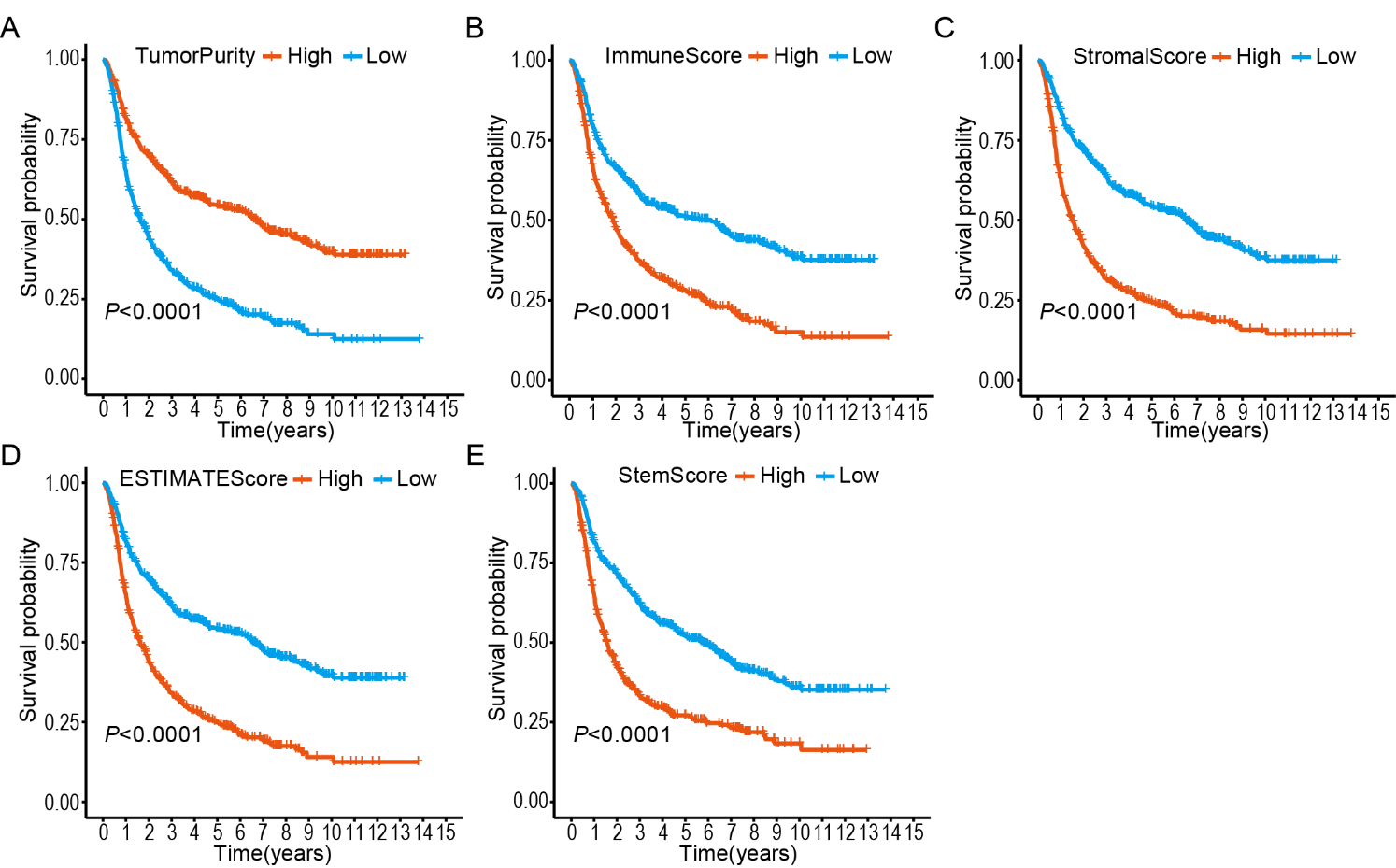


**Figure S3.** The prognostic value of immune score and stemness score. Kaplan–Meier overall survival curve for tumor purity patients (A), Immune score (B), Stromal score (C), ESTIMATE score (D), and Stemness score (E).


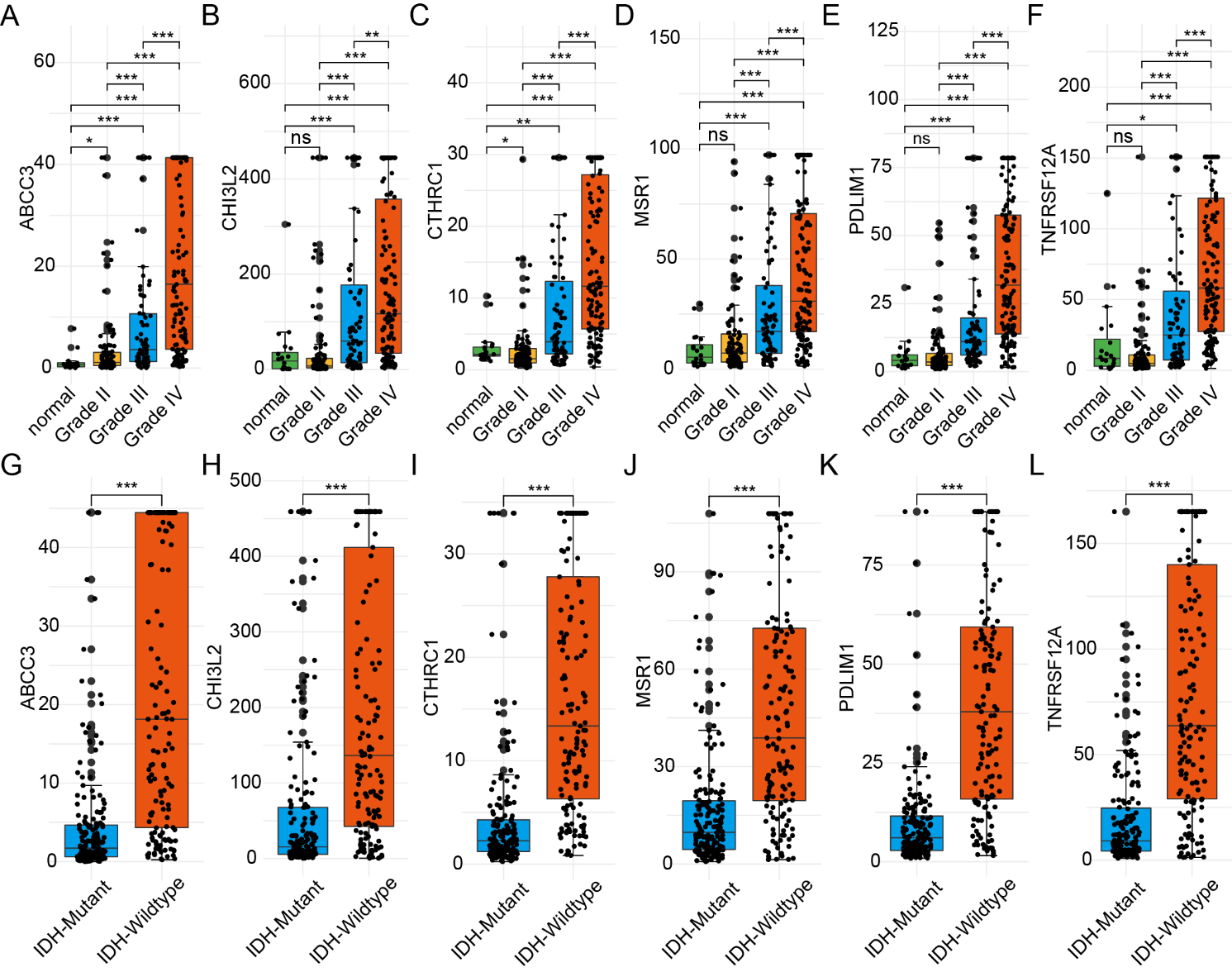


**Figure S4.**  Expression of six-gene in gliomas with different grade and IDH status. ***$\text{ P}$ < 0.001; **$\text{ P}$ < 0.01; *$\text{ P}$ < 0.05; ns, no significant.


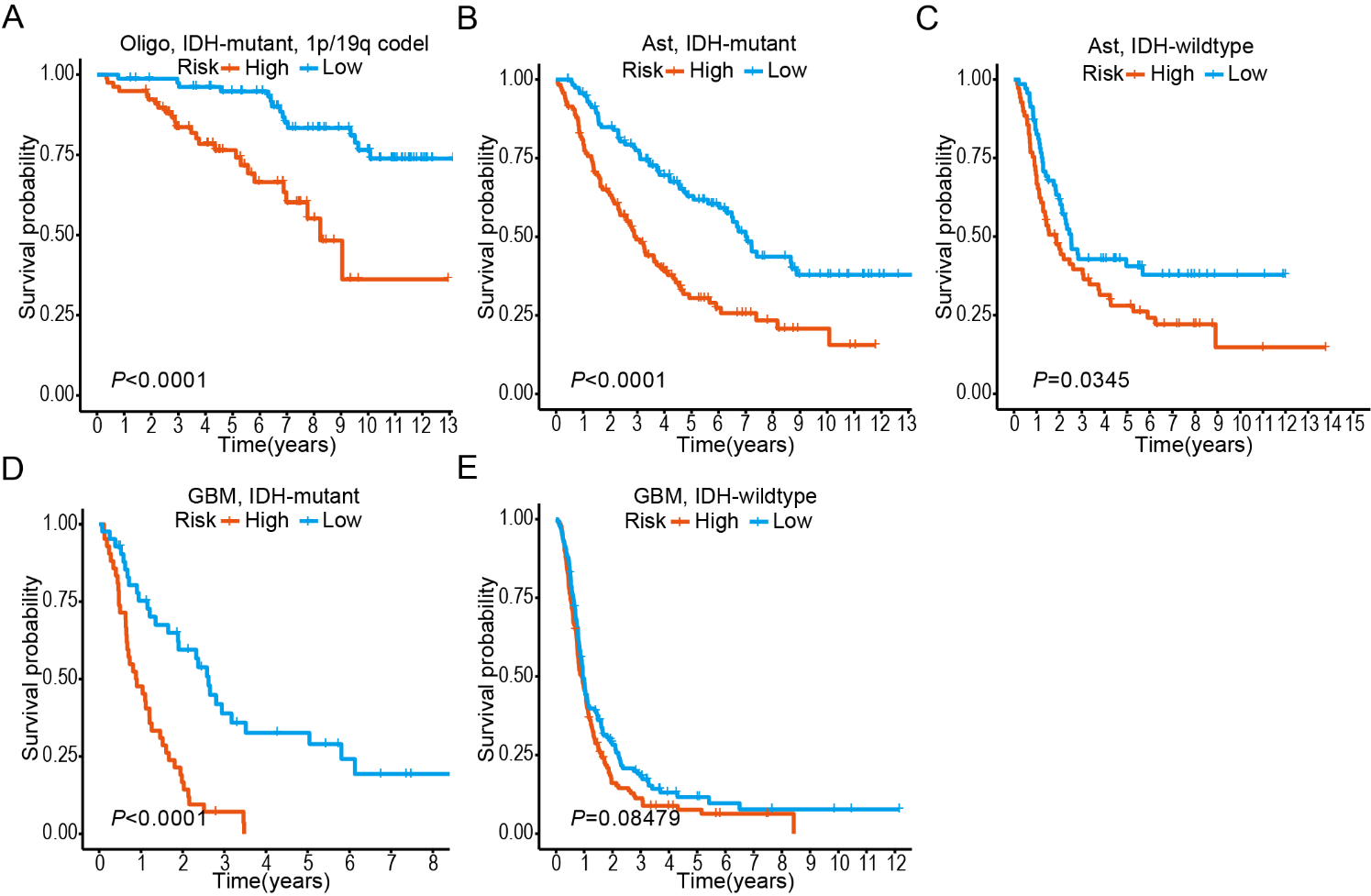


**Figure S5.** Prognostic values of the risk signature in patients stratified by the integrated analysis of WHO 2016. (A-E) Kaplan–Meier overall survival curves for patients with Oligodendroglioma with IDH-mutant and 1p/19q co-deletion (A), Astrocytoma with IDH-mutant (B), Astrocytoma with IDH-wildtype (C), GBM with IDH-mutant (D), and GBM with IDH- wildtype (E).


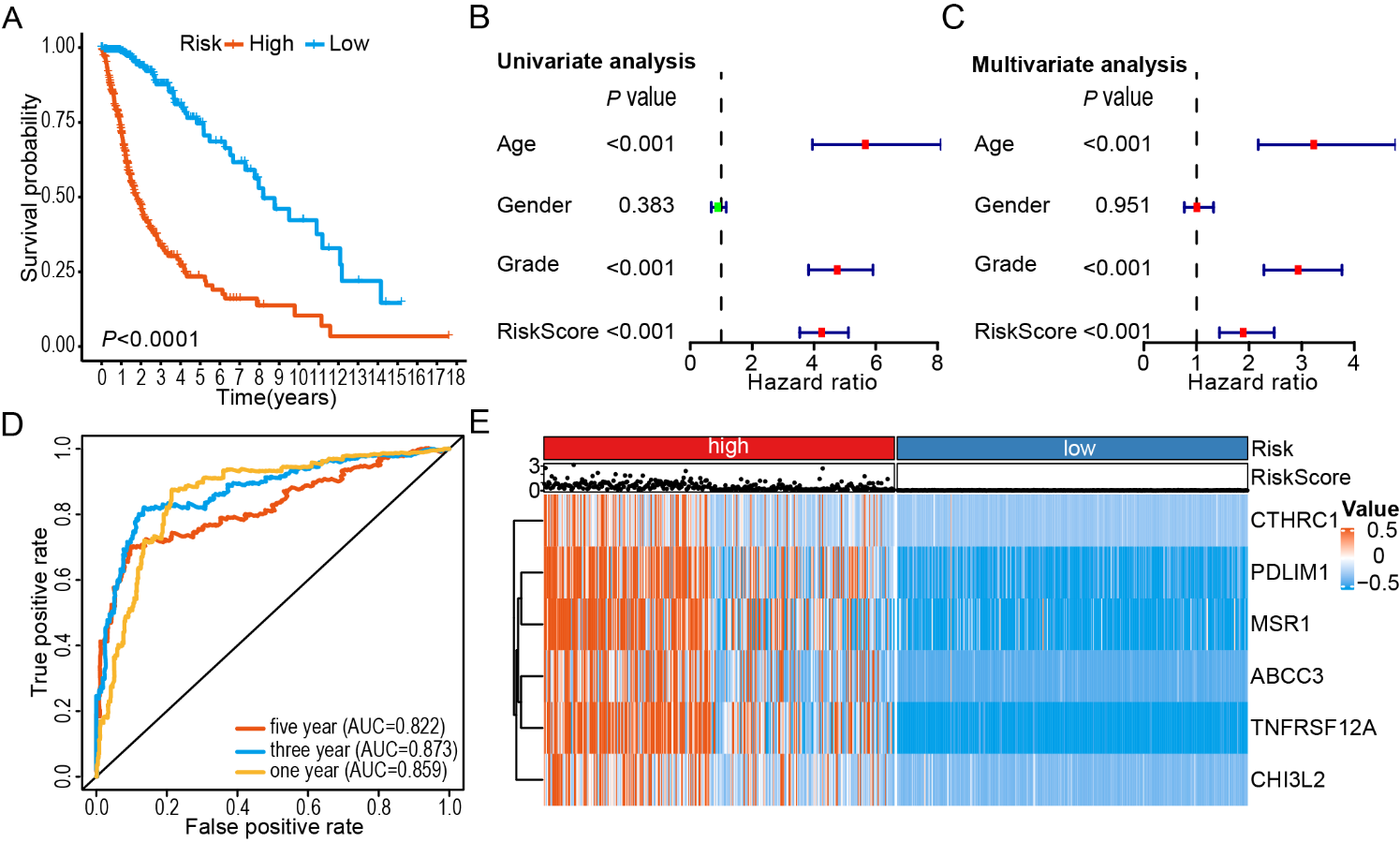


**Figure S6.** Prognostic significance of risk signature-derived risk scores in the TCGA cohort. (A) Patients with TCGA gliomas were analyzed by Kaplan-Meier analysis and stratified by median risk. A high-risk score is often associated with a lower survival rate for gliomas. (B) Univariate Cox regression analysis. Forest map of the association between risk factors and glioma survival. (C) Multivariate Cox regression analysis. Risk signature is an independent predictor of gliomas. (D) ROC curves were used to measure the predictive value of the risk score. (E) In the TCGA cohort, six genes were expressed in high-risk population.


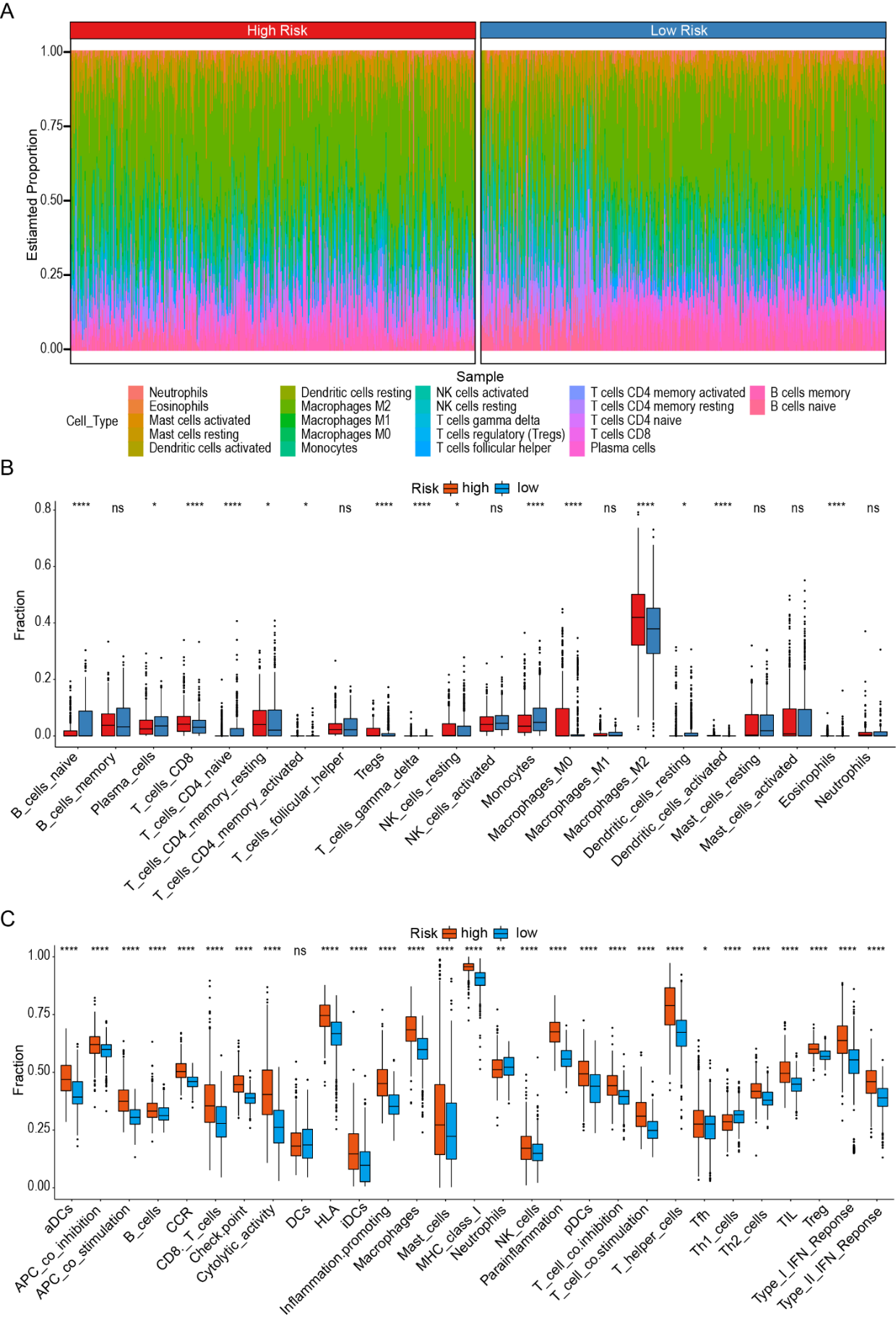


**Figure S7**. Differences of immune cell infiltration among risk subtypes. (A) The mean proportion of 22 immune cells in the CGGA cohort. (B-C) Different algorithms were used to calculate the expression of immune cells in the high and low-risk subtypes in the CGGA cohort: CIBERSORT (B) and ssGSEA (C). ****$\text{ P}$ < 0.0001; **$\text{ P}$ < 0.01; *$\text{ P}$ < 0.05; ns, no significant.
